# Deficiency of Wnt10a causes female infertility via the β-catenin/Cyp19a1 pathway in mice

**DOI:** 10.7150/ijms.71127

**Published:** 2022-03-28

**Authors:** Jia-He Zhang, Takashi Tasaki, Manabu Tsukamoto, Ke-Yong Wang, Kagaku Azuma

**Affiliations:** 1 Department of Anatomy, School of Medicine, University of Occupational and Environmental Health, 1-1 Iseigaoka, Yahatanishiku, Kitakyushu 807-8555, Japan.; 2 Department of Pathology, Kagoshima University Graduate School of Medical and Dental Sciences, 8-35-1 Sakuragaoka, Kagoshima, 890-8544, Japan.; 3 Department of Orthopaedic Surgery, School of Medicine, University of Occupational and Environmental Health University, 1-1 Iseigaoka, Yahatanishi-ku, Kitakyushu 807-8555, Japan.; 4 Shared-Use Research Center, School of Medicine, University of Occupational and Environmental Health, 1-1 Iseigaoka, Yahatanishi-ku, Kitakyushu 807-8555, Japan.

**Keywords:** Wnt10a, Female fertility, Sex hormone, Cyp19a1, Ovarian follicle, Endometrium

## Abstract

Wnt signaling is relevant for a wide range of biological processes, including reproductive function. The function of Wnt10a in female fertility, however, remains obscure. In the present study, we explored the structure and function of the female reproductive organs in Wnt10a knockout (KO) mice. The expression of β-catenin signaling was significantly lower in the ovaries of the Wnt10a KO mice compared with wild-type (WT) mice. In addition, the estrous cycles were disrupted, ovarian follicles were diminished, and endometria were thinner, accompanied by lower serum estrogen levels, and higher testosterone and progesterone levels in Wnt10a KO mice. The expression of the ovarian cytochrome P450 family 19 subfamily A member 1 (Cyp19a1) was significantly lower in Wnt10a KO mice. We detected no significant changes in the levels of the gonadotropins between WT and KO mice. Together, our findings indicate that deficiency of Wnt10a causes female infertility through β-catenin and Cyp19a1signaling pathways in mice.

## Introduction

Mammalian female reproductive functions depend on tight regulation of the hypothalamic-pituitary-gonadal axis 1, 2. Gonadotropin-releasing hormone (GnRH), produced and secreted by the hypothalamus, acts on the pituitary gland to stimulate the synthesis and secretion of gonadotropins, follicle-stimulating hormone (FSH) and luteinizing hormone (LH). FSH and LH, in turn, act through their receptors on ovarian follicles, promote follicular growth and maturation, and stimulate the production of estrogen and progesterone. Interactions among these hormones and their receptors are vital for maintaining female fertility. Disruptions of the hormonal communication can lead to infertility 1, 2.

A woman's fertility depends upon the timely and appropriate orchestration of the ovaries, uterus, and their functions. Dysregulation of the signaling pathways involved in female reproduction leads to difficulties in becoming pregnant. Infertility in humans is defined as the failure to achieve pregnancy during 12 months of regular sexual intercourse. The prevalence of female infertility is drastically increasing, affecting approximately 48 million women throughout the world, and is often associated with significant physical and emotional changes 3. Polycystic ovary syndrome (PCOS), which is associated with hyperandrogenism, irregular cycles, and other several disorders, is the most common cause of female infertility. The precise pathogenesis of PCOS remains poorly understood. Emerging evidence indicates that the Wnt/β-catenin signaling pathway in the ovarian granulosa cells may be involved in the pathogenesis of PCOS 4.

The ovaries are a pair of female organs in the pelvis. The primary function of the ovaries is to generate and release oocytes regularly, and to produce steroid hormones. The ovarian follicle, composed of different numbers and types of cells, is the basic unit of the ovary and provides the environment for protecting and nourishing the oocyte. Ovarian follicles growth and maturation are controlled by well-orchestrated factors, including the pituitary FSH, LH, various cytokines, and several local signaling pathways 1, 5. Estrogen is very important for regulating female reproductive functions. Aromatase cytochrome P450 (Cyp) is a large enzymatic protein superfamily with important roles in the synthesis and metabolism of many signaling molecules, particularly steroid hormones 6. Cyp11a1 converts cholesterol from follicular theca cells into progesterone 7. Progesterone is subsequently transformed into androgen under the actions of Cyp17a1. Conversion of androgen to estrogen is catalyzed by Cyp19a1, which is a critical enzyme in estrogen synthesis. Circulating estrogen levels are regulated by the pituitary FSH, which modulates the transcription of Cyp19a1 in the ovarian follicular granulosa cells 8, 9.

Accumulating evidence reveals a critical role of the Wnt signaling pathway in maintaining female fertility 10-14. Wnt signaling is relevant for a wide range of cellular processes, including cell proliferation, differentiation, migration, and cell fate 15. There are 3 known Wnt signaling pathways: canonical β-catenin-dependent, non-canonical planar cell polarity, and non-canonical Wnt/calcium pathways. The canonical Wnt/β-catenin pathway has an essential role in regulating cell behavior and gene expression. The non-canonical pathways are involved in cell polarity and the regulation of intracellular calcium levels. Wnt/β-catenin signaling pathway has been recognized to contribute to ovarian regulation of steroidogenesis, as β-catenin is an essential transcriptional modulator of Cyp19a1 8.

The Wnt10a is part of a large family of Wnt genes. Wnt10a gene mutations were first identified in odonto-onycho-dermal dysplasia, affecting the teeth, nails, and skin 16. We recently found that Wnt 10a also plays a critical role in wound healing, cancer growth, osteogenesis, and adipogenesis 17-19. No studies to date, however, have evaluated the association between Wnt10a and female fertility. Here, we provide novel evidence that Wnt10a signaling is involved in female fertility potentially through β-catenin and its associated downstream factor, Cyp19a1 by using wild-type (WT) and Wnt10a knockout (KO) mice.

## Materials and methods

### Animals

Wnt10a KO mice were generated as described previously 19. Experiments were performed in 8-, 12-, 16-, 20-, and 24-week-old female Wnt10a KO mice and age-matched female WT (C57BL/6J) mice (Charles River, Yokohama, Japan). The transgenic experiment was evaluated and approved by the local committee (DP150019C3, August 12, 2015). Mice were housed in the Laboratory Animal Research Center at the University of Occupational and Environmental Health, Japan. Animals were provided with standard mouse chow and drinking water ad libitum and maintained on a 12-h light/dark cycle. All animal experimental protocols were approved by the Ethics Committee for Animal Care and Use of the University of Occupational and Environmental Health, Japan (AE-18-029, February 14, 2019).

### Estrous cycle determination

Daily vaginal smears from 12-week-old mice were collected with cotton swabs at the same time each morning (8:00-9:00 a.m.) for 12 successive days. Dried smears were stained with Giemsa stain, examined microscopically, and the estrous cycle stage was classified according to established criteria 20. When the mouse is in proestrous, mostly nucleated and some cornified epithelia are present. In the estrous stage of the cycle, mostly cornified epithelia are present. During diestrous, primarily polymorphonuclear leukocytes and a few epithelia are present.

### Histopathological observations

The WT and Wnt10a KO mice were killed by intraperitoneal injection of a mixture of ketamine 50 mg/kg and medetomidine 1 mg/kg 19. Mouse ovaries and uteri were dissected, and the weights were measured using an electronic analytical balance. The ovarian and uterine tissues were fixed with 10% formalin and embedded in paraffin. Sections were cut to 4-μm thickness and stained with hematoxylin and eosin. All sections were observed with a light microscope (Olympus VS120, Olympus, Tokyo, Japan) and digitized with software VS-ASW (Olympus). The ovarian follicles were classified into 4 types as described previously 21, 22: primordial follicles, having one layer of flattened pre-granulosa cells surrounding the oocyte; primary follicles, showing one layer of cuboidal granulosa cells; secondary follicles, containing at least two layers of granulosa cells; and atretic follicles, exhibiting disorganized, apoptotic, and degenerative changes in the eosinophilic granulosa cells of the ooplasm, contraction and clumping of the chromatin, and wrinkling of the oocyte nuclear membrane. The endometrial thickness was measured from the luminal surface to the beginning of the circular smooth muscle layer with ImageJ software, as previously reported 2.

### Immunohistochemical observation

Paraffin sections of the mouse ovaries and uterus were deparaffinized in xylene and rehydrated in an ascending series of ethanol solution. After antigen retrieval, sections were treated with blocking solution. Sections were incubated with anti-β-catenin (1:1000, Santa Cruz, Dallas, TX, USA), anti-cytochrome P450 family 19 subfamily a member 1 (Cyp19a1, 1:100, Assay Biotechnology Company, Inc., Sunnyvale, CA, USA), and anti-Wnt10a (1:5000). For generating anti-Wnt10a antibody, rabbits were immunized with a synthetic peptide corresponding to amino acids 160-172 of the mouse Wnt10a. The sera of the immunized rabbit were then isolated and purified 19. Sections were then incubated with biotinylated goat anti-rabbit IgG and streptavidin peroxidase complex (Nichirei Biosciences Inc., Tokyo, Japan), stained with diaminobenzidine and then counterstained with hematoxylin. Images were captured with a light microscope (Olympus VS120) and digitized with software VS-ASW (Olympus).

### ELISA Assay

Blood samples were collected at 12 weeks of age and centrifuged at 1500 ×g for 10 min at 4 °C to separate the serum. The serum concentrations of 17β-estradiol, testosterone, progesterone, FSH, and LH were measured using enzyme-linked immunosorbent assay kits, in accordance with the manufacturer's instructions (Enzo Life Sciences, Inc., Farmingdale, New York, USA). The absorbance of light was determined with a microplate reader (Corona Electric Co Ltd., Ibaraki, Japan).

### Real-time PCR

Total RNA was extracted from the ovarian tissues at 12 weeks of age with Trizol reagents (Invitrogen). First-strand cDNA was synthesized with Superscript II RT (Invitrogen) in accordance with the manufacturer's instructions. The cDNA was applied to evaluate mRNA expression levels of Wnt10a, β-catenin, FSH receptor (FSHR), steroidogenic factor 1 (Sf1), and GAPDH in the ovarian tissue. The following specific primers used: Wnt10a (Mm00437325_m1, Applied Biosystems, Foster City, CA, USA), β-catenin (Mm00483039_m1, Applied Biosystems), FSHR (Mm00442819_m1, Applied Biosystems), Cyp19a1 (Mm00484049_m1, Applied Biosystems), Sf1 (Mm00496060_m1, Applied Biosystems), and GAPDH (Mm99999915_g1, Applied Biosystems). Quantitative real-time polymerase chain reaction (PCR) analysis was performed using the TaqMan fluorogenic probe method with a Step One real-time PCR system (Thermo Fisher Scientific, Waltham, MA, USA). Cycling conditions were as follows: 50 °C for 2 min, 95 °C for 10 min followed by 45 cycles of 95 °C for 15 s and 60 °C for 1 min. The mRNA expression levels were normalized with the GAPDH mRNA expression level.

### Western blot analysis

Ovarian tissues of WT and Wnt10a KO mice at 16 weeks of age were lysed in RIPA (Millipore corp., Bedford, MA, USA), centrifuged at 12,000× g for 30 min, and the supernatants were collected for analysis. The proteins were separated using sodium dodecyl sulfate-polyacrylamide gel electrophoresis and transferred to Immun-Blot polyvinylidene difluoride membranes (Bio-Rad Laboratories, K.K., Tokyo, Japan) with a semi-dry blotter. The blotted membranes were treated with 5% skim milk in 10 mM Tris, 150 mM NaCl and 0.2% Tween-20 and incubated for 1 h at room temperature with the primary antibodies anti-Cyp19a1 (58 kDa, 1:1000, Assay Biotechnology Company, Inc.) and anti-GAPDH (37 kDa, 1:1000, Cell Signaling Technology, Inc., Danvers, MA, USA). The immunoblotting membranes were then incubated with a peroxidase-conjugated secondary antibody (1:1000, Cell Signaling Technology) for 60 min and visualized with an ECL kit (GE Healthcare Bio-Science, Buckinghamshire, UK). Bands of the target proteins were analyzed using the Scion Image software program (version 4.0.2; Scion Corp., Frederick, MD, USA).

### Microarray assay

DNA microarray analyses were performed using 3D-Gene (Toray Industries, Kamakura, Kanagawa, Japan), as described previously 17. RNA samples were extracted from ovarian tissues of 12-week-old WT and Wnt10a KO mice, and labeled with Cy3-CTP and Cy5-CTP (Amersham Biosciences Corp., Piscataway, NJ, USA), by using low RNA input fluorescent linear amplification kits (Agilent Technologies, Inc., Santa Clara, CA, USA) according to the manufacturer's instructions. The dye incorporation ratio was measured with a Nanodrop spectrophotometer. For hybridization, Cy3-labeled and Cy5-labeled cRNA were fragmented and hybridized to Agilent Gene Expression Microarray, as described under Two-color Microarray-based Gene Expression Analysis (Agilent Technologies, Inc.). The hybridized arrays were scanned and the resulting images were analyzed by the Rosetta Resolver1 software program (Rosetta Biosoftware, Kirkland, WA, USA). The datasets were deposited in NCBI's Gene Expression Omnibus (accession number GSE23969). All samples were evaluated in triplicate. Genes were selected as significant using the criterion of more than two folds of up- or down-regulated changes.

### Statistical analysis

The experimental data are expressed as means ± standard deviation (SD), and an unpaired *t*-test was applied to analyze differences between the KO and WT mice, using SPSS software (version 22.0, Chicago, IL, USA). The difference is considered to be statistically significant when p values < 0.05.

## Results

### Wnt10a KO mice showed estrous cycle disruption

**Figure 1:**
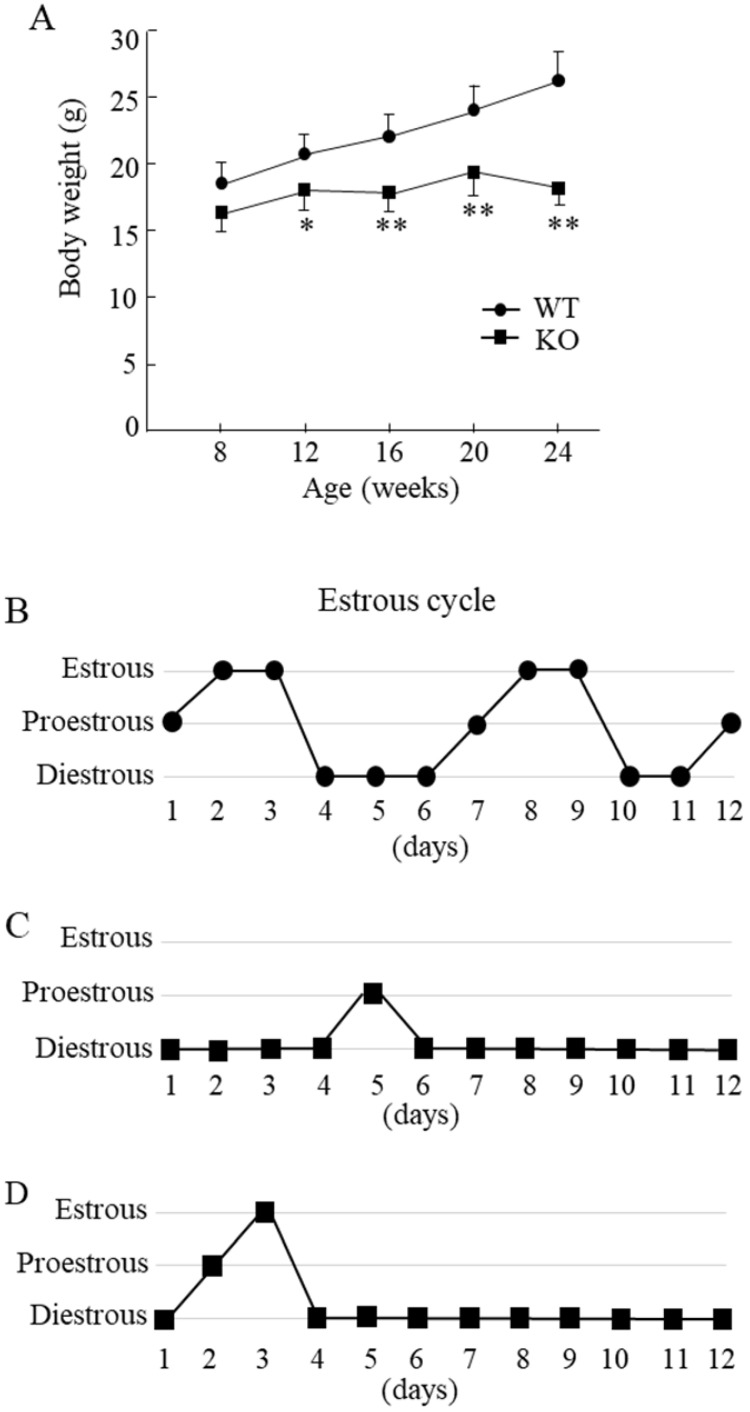
** Body weight and the estrous cycle in WT and Wnt10a KO mice.** Body weights were significantly lower in KO mice from 12 to 24 weeks of age **(A)**. Data are expressed as the mean ± SD. n=7 mice/group. *p < 0.05; ** p < 0.01. WT mice showed a regular 5-day estrous cycle in the chronological order of proestrous, estrous, and diestrous **(B)**. Wnt10a KO mice showed a disrupted estrous cycle, characterized by an extended diestrous stage **(C, D)**.

Compared with the WT mice, Wnt10a KO mice were smaller in size. The Wnt10a KO mice also tended to have lower body weights at 8 weeks of age. From 12 to 24 weeks of age, the body weights were significantly lower in Wnt10a KO mice (Fig. 1A), indicating the growth retardation. Microscopic evaluation of the vaginal smears obtained over 12 successive days was used to verify stages of the estrous cycle. The WT mice showed a regular 5-day estrous cycle in the chronological order of proestrous, estrous, and diestrous (Fig. 1B). Wnt10a KO mice showed disrupted estrous cycle, characterized by increased time spent in diestrous (Fig. 1C, D). We did not detect the estrous stage in some Wnt10a KO mice.

### Wnt10a KO mice exhibited decreased ovarian reserve and function, and thinner endometrium

**Figure 2:**
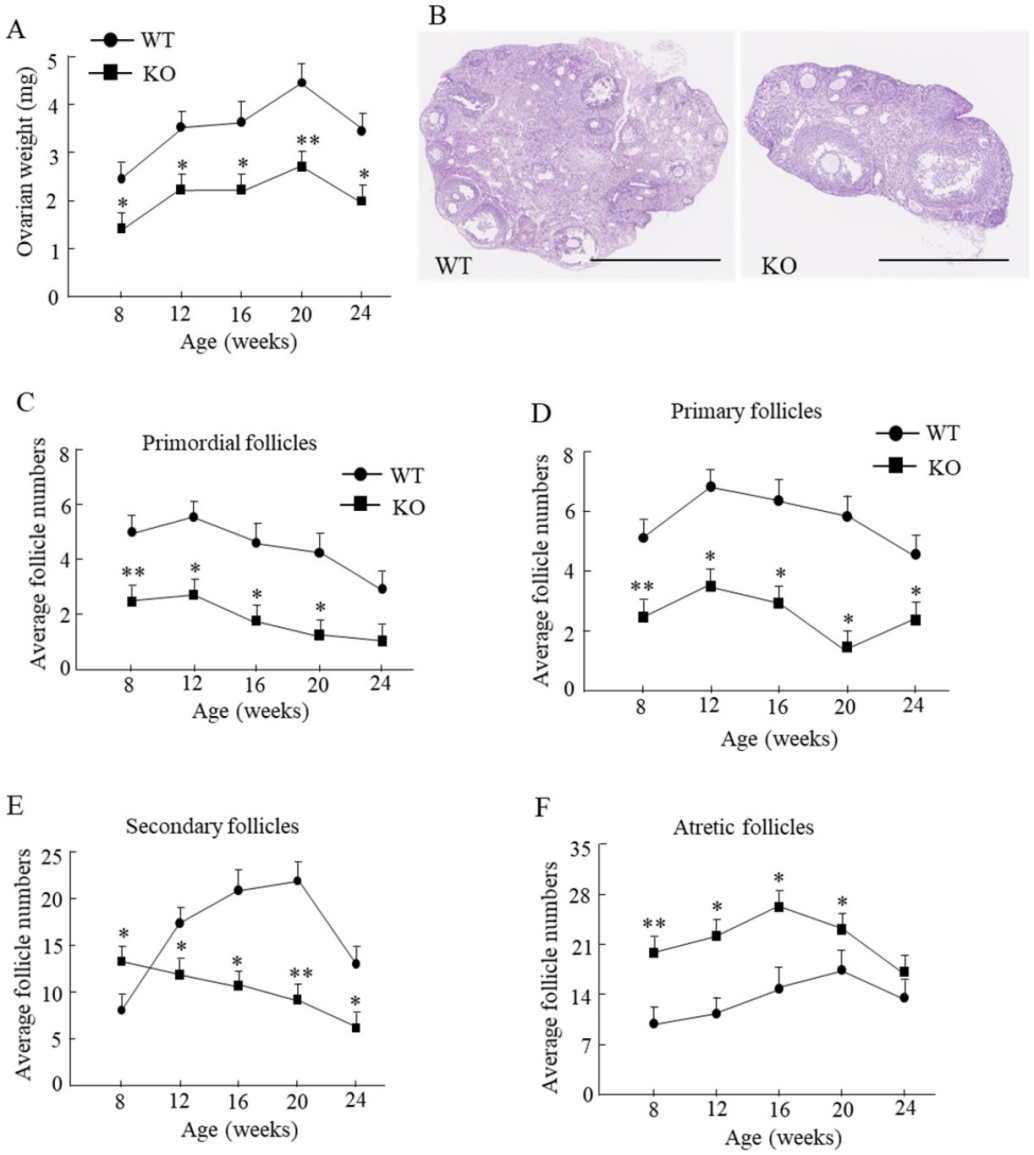
** Ovarian weight and the ovarian follicles in WT and Wnt10a KO mice.** Ovarian weight was significantly lower in Wnt10a KO mice **(A)**. n=7 mice/group. *p < 0.05; ** p < 0.01. Representative ovary hematoxylin and eosin staining in 12-week-old WT and Wnt10a KO mice **(B)**. Scale bars=1.0 mm. Primordial follicle numbers were significantly reduced in Wnt10a KO mice from 8 to 20 weeks **(C)**. Primary follicle numbers were significantly diminished in Wnt10a KO mice from 8 to 24 weeks **(D)**. Secondary follicle numbers were decreased in 8-week-old Wnt10a KO mice, and increased in Wnt10a KO mice from 12 to 24 weeks **(E)**. Atretic follicle numbers were significantly increased in Wnt10a KO mice from 8 to 20 weeks **(F)**. Data are expressed as the mean ± SD.

Compared with the WT mice, the ovarian weight in Wnt10a KO mouse was significantly lower from 8 to 24 weeks of age (Fig. 2A). We tested the ovarian reserve and function by determining the number of ovarian follicles microscopically (Fig. 2B). Primordial follicles were significantly decreased in Wnt10a KO mice from 8 to 20 weeks of age (Fig. 2C). Primary follicles were significantly decreased in Wnt10a KO mice from 8 to 24 weeks of age (Fig. 2D). Secondary follicles were significantly higher in 8-week-old Wnt10a KO mice and significantly lower in Wnt10a KO mice compared with WT mice from 12 to 24 weeks of age (Fig. 2E). Atretic follicles were significantly increased in Wnt10a KO mice compared with WT mice from 8 to 20 weeks of age (Fig. 2F), indicating the decreased ovarian reserve and function in Wnt10a KO mice.

**Figure 3:**
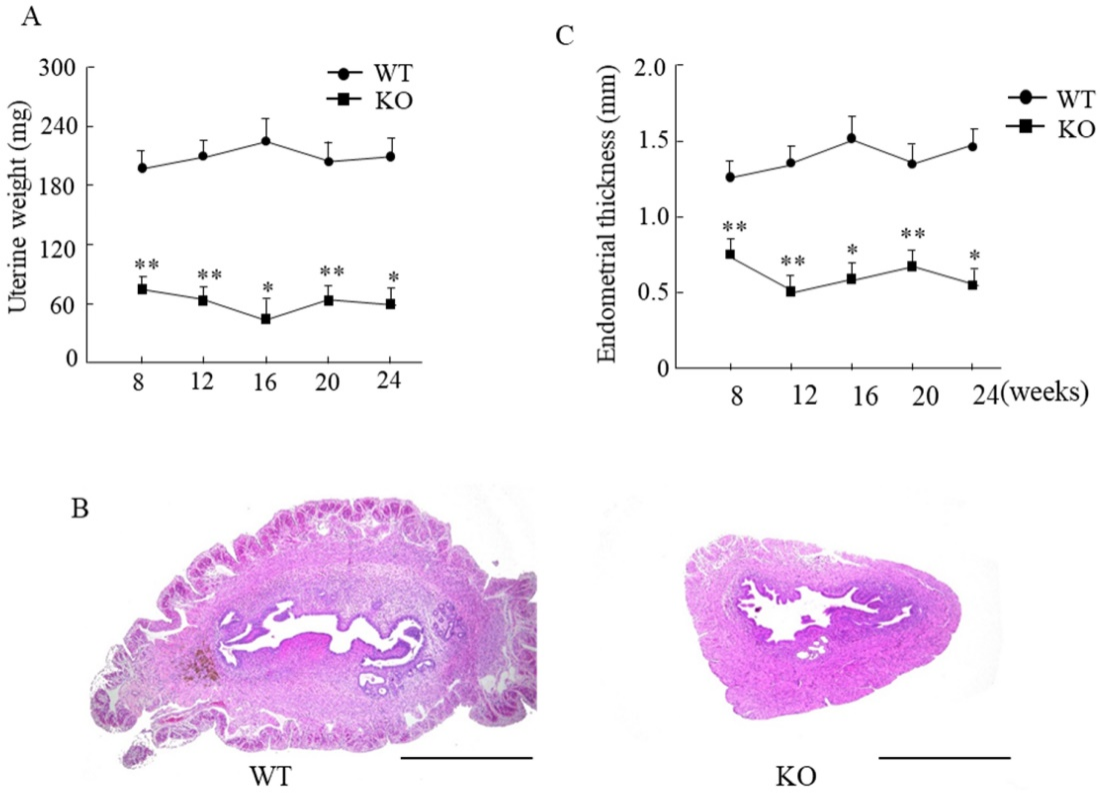
** Uterine weight and the endometrial thickness in WT and Wnt10a KO mice.** Uterine weight was significantly lower in Wnt10a KO mice **(A).** Data are expressed as the mean ± SD. n=4-6 mice/group. *p < 0.05; ** p < 0.01. Representative uterine hematoxylin and eosin staining in 12-week-old WT and Wnt10a KO mice **(B).** Scale bars=1.0 mm. Endometrium was significantly thinner in Wnt10a KO mice **(C).** n=4-6 mice/group. *p < 0.05; ** p < 0.01.

The uterine weight in Wnt10a KO mouse was significantly lower than that in WT mouse (Fig. 3A). The endometrium was significantly thinner in Wnt10a KO mice than in WT mice from 8 to 24 weeks of age (Fig. 3B, C). The presence of endometrial glands was confirmed in both WT and Wnt10a mice.

### Wnt10a KO mice exhibited lower expression of ovarian β-catenin

**Figure 4:**
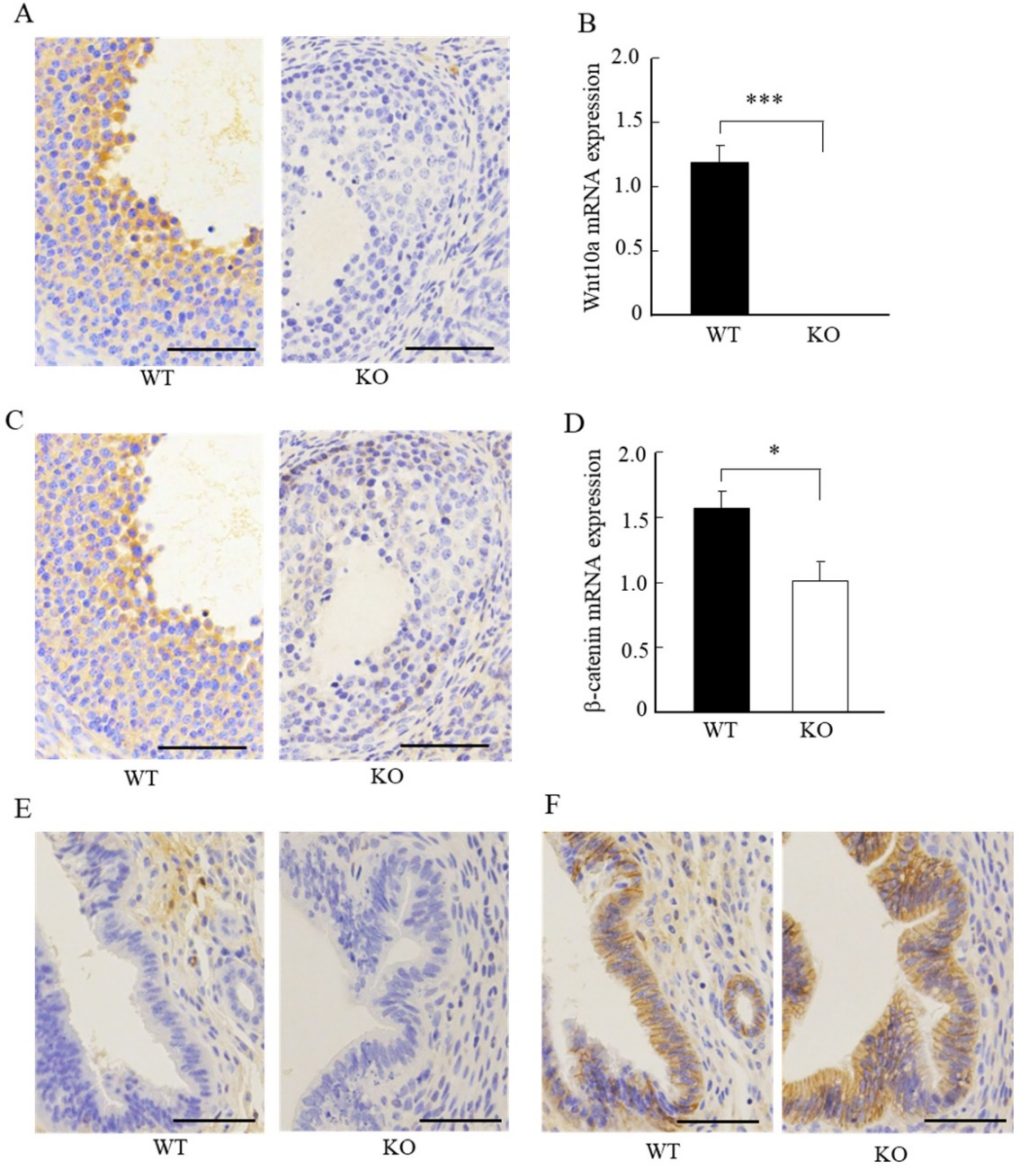
Representative immunohistochemical images of Wnt10a and β-catenin in mouse ovaries and uterus. Wnt10a-positive cells were present in the ovary of WT mouse. There were no Wnt10a-positive cells in the ovary of Wnt10a KO mouse **(A)**. Expression of Wnt10a mRNA was not detected in Wnt10a KO mouse ovary **(B)**. Compared with WT mice, β-catenin-positive cells were decreased in ovary follicular granulosa cells of Wnt10a KO mice **(C)**. The mRNA expression of ovarian β-catenin was significantly lower in Wnt10a KO mice than in WT mice **(D)**. Wnt10a-positive cells were present in the uterus of WT mouse. There were no Wnt10a-positive cells in the uterus of Wnt10a KO mouse **(E)**. β-Catenin-positive cells were present in the uterine epithelium of both WT and Wnt10a KO mice **(F)**. Scale bars=0.1 mm. Data are expressed as the mean ± SD. *p < 0.05; ***p < 0.001.

We next examined the expression of Wnt10a and β-catenin signaling in the ovaries and uteri by immunohistochemistry. Wnt10a-positive cells were present in ovaries of the WT mice. There were no Wnt10a-positive cells in the KO mice (Fig. 4A), and no Wnt10a mRNA expression in Wnt10a KO mouse ovaries (Fig. 4B). Compared with WT mice, β-catenin-positive cells were decreased in the follicular granulosa cells of KO mice (Fig. 4C). Quantitative PCR analysis showed that the β-catenin mRNA expression level was significantly lower in Wnt10a KO mice than in WT mice (Fig. 4D).

Wnt10a-positive cells were present in the uterus of the WT mice. We did not detect any Wnt10a-positive cells in the KO mice (Fig. 4E). The β-catenin-positive cells were present in the endometrial glandular and stromal cells of both WT and KO mice (Fig. 4F). No significant differences in the number of β-catenin-positive cells in the uterus were detected between WT and Wnt10a KO mice (Fig. 4F).

### Wnt10a KO mice displayed low estrogen, and high testosterone and progesterone levels

**Figure 5:**
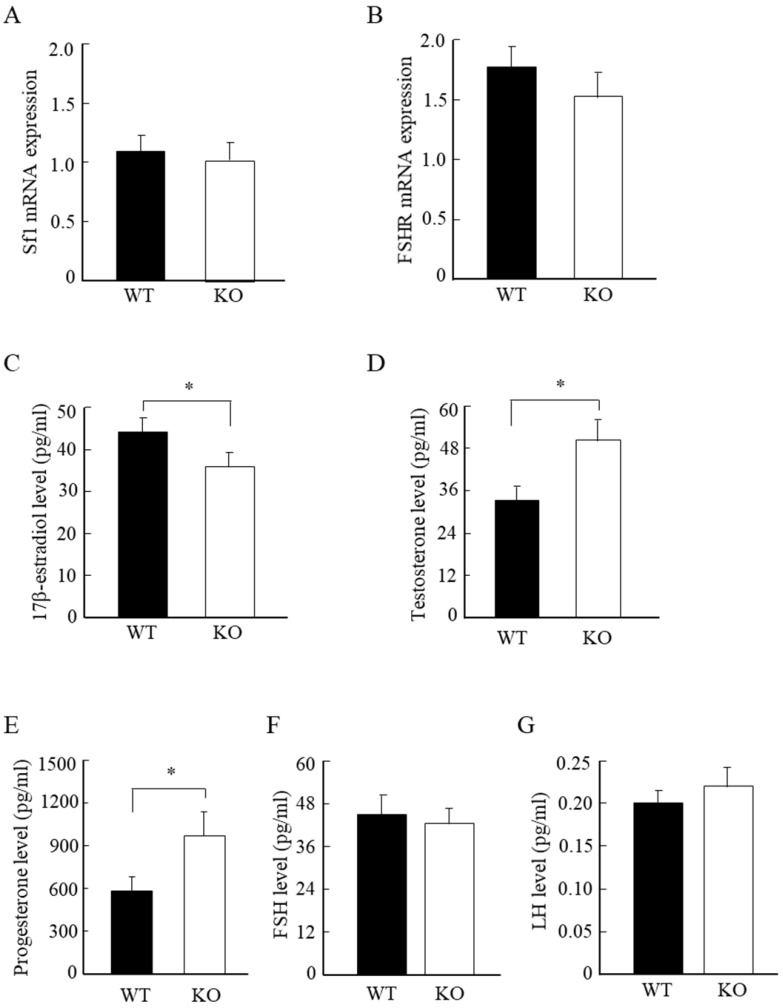
** The mRNA expression of Sf1 and FSHR, and the serum hormone levels in WT and Wnt10a KO mice.** There were no significant differences in the mRNA expression levels of Sf1 and FSHR between WT and Wnt10a KO mice **(A, B)**. The serum 17β-estradiol level was significantly lower in Wnt10a KO mice **(C)**. The serum testosterone and progesterone levels were significantly higher in Wnt10a KO mice **(D, E)**. There were no significant differences in the serum FSH and LH levels between WT and Wnt10a KO mice (F, G). n=5 mice/group. Data are expressed as the mean ± SD. *p < 0.05.

To further analyze the molecular changes in the murine ovaries, we examined the mRNA expression levels of ovarian Sf-1 and FSHR. We detected no significant differences in the mRNA expression levels of ovarian Sf-1 and FSHR between WT and Wnt10a KO mice (Fig. 5A, B). We also measured the serum steroid hormone levels by ELISA assay. The serum 17β-estradiol concentration was significantly lower in Wnt10a KO mice than in WT mice (Fig. 5C), whereas the serum levels of testosterone and progesterone were significantly higher in Wnt10a KO mice (Fig. 5D, E). There were no significant differences in the serum levels of FSH and LH levels between WT and Wnt10a KO mice (Fig. 5F, G).

### Wnt10a KO mice exhibited downregulation of ovarian Cyp19a1

**Figure 6:**
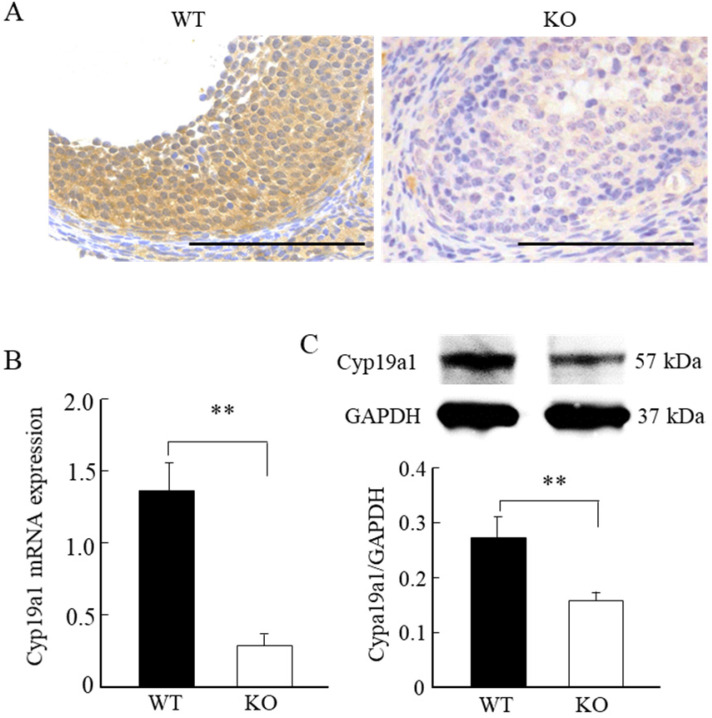
** Cyp19a1 expression in the ovaries of 8-week-old WT and Wnt10a KO mice.** Representative immunohistochemical images of Cyp19a1-positive ovarian granulosa cells (A). Scale bars=0.1 mm. Compared with the WT mice, the Cyp19a1mRNA and protein expression levels in the ovaries were significantly lower in Wnt10a KO mice (B, C). Data are expressed as the mean ± SD. **p < 0.01.

Immunohistochemical staining, quantitative PCR, and Western blot analyses revealed significantly lower levels of Cyp19a1 mRNA and protein expression levels in the ovarian follicular granulosa cells of the Wnt10a KO mice compared with WT mice (Fig. 6A-C).

The cDNA microarray analyses were performed to compare the ovarian tissues of the WT and Wnt10a KO mice. We evaluated a total of 11 genes potentially involved in female fertility and found the apparent downregulation of Wnt10a, Cyp19a1, and LH/choriogonadotropin receptors (Lhcgr) in Wnt10a KO mice (Table 1).

## Discussion

The present study revealed that Wnt10a KO mice had prolonged and disrupted estrous cycles, decreased ovary size and the number of ovarian follicles, thinner uterine endometrium, lower serum estrogen levels, and higher testosterone and progesterone levels, reflecting the female reproductive dysfunction.

The periodicity of estrous cycle in mature females is a direct result of the cyclic alterations of the female reproductive functional status. During estrous cycle, the circulating levels of LH, FSH, estrogen, and progesterone exhibit rhythmic alterations 23. In this study, we found that the WT mouse showed a regular 5-day estrous cycle, indicating the normal ovary function. Wnt10a KO mouse exhibited irregular estrous cyclicity, characterized by prolonged cycle length with longer diestrous stage, suggesting disrupted female reproductive function.

The ovarian follicle, composed of different numbers and types of cells, is the basic unit of the ovary, and provides the environment for protecting and nourishing the oocyte. Ovarian follicular development is a complex dynamic process, which is controlled by well-orchestrated factors, including the pituitary FSH, LH, various cytokines, and several intracellular signaling pathways 1, 5. The ovarian follicles are recruited from the primordial follicle for further growth and maturation in a continuous fashion. Cyclic follicular recruitment in mice is characterized by various hormonal and physiological alterations 20. Several studies have reported that the expression of multiple Wnt components in ovarian follicles. β-Catenin is the principal molecule in the canonical Wnt/β-catenin signaling pathway and might facilitate the effects of FSH on ovarian follicular cells and promote proliferation of the granulosa cells 14. Our results showed that the ovary weight was much lower and the number of ovarian primordial, primary, and secondary follicles was significantly decreased in Wnt10a KO mouse, indicating the decreased ovarian reserve and function. Forkhead transcription factor subfamily O1 (FoxO1), a transcription factor, is a key regulator of cellular homeostasis, including cell proliferation and cell death. Accumulation of the nuclear FoxO1 in follicular granulosa cells was associated with increased follicle atresia 24. Wnt/β-catenin signaling pathway can regulate FoxO1 cellular localization, decreasing the nuclear FoxO1 levels 25. We speculate that deletion of Wnt10a increased the nuclear FoxO1 level, leading to follicular atresia. In this study, we found that the number of atretic follicles was significantly higher in Wnt10a KO mouse.

The endometrium is a highly dynamic tissue and undergoes periodic alterations morphologically and functionally. Several kinds of Wnt receptors are expressed in the endometrium or stroma of the uterus 26, 27. Wnt/β-catenin signaling pathway plays critical roles in maintaining the uterine endometrial homeostasis for fertility. Conditional ablation of Wnt7a in the mouse uterus inhibits endometrial gland development, and causes infertility due to the inability of the uterus to support embryo implantation 26, 27. The activation of Wnt signaling is to promote the endometrial gland formation. The uterine Wnt signaling of different compartments showed to interact with each other. A single gene deletion of Wnt may be sufficient to inhibit endometrial gland formation 28. The deletion of Wnt signaling in uterus manifests normal postnatal initial development of the endometrial glands, but induces disturbance of the glands during adulthood 29. As an important indicator of endometrial receptivity, endometrial thickness below a certain threshold would affect pregnancy 30. Our results demonstrated that the uterine weight was lower and endometrium became thinner, with no significant changes of β-catenin expression in the uterus of Wnt10a KO mice, suggesting postpubertal endometrial dysfunction and infertility.

Ovarian follicular granulosa cells are key cells in steroid hormone production and secretion. β-Catenin is an essential transcriptional regulator of Cyp19a1, activating the expression of Cyp19a1 in the granulosa cells through the functional interactions with Sf1 31. The transcription factor Sf1, encoded by nuclear receptor family 5 group A member 1 (NR5A1), plays a crucial role in the development and function of the adrenal glands and reproductive tissues at multiple levels 32. It is also essential for hypothalamic and pituitary function. The expression of Sf1 can be modulated by β-catenin signaling 9. Stimulation of β-catenin signaling downregulates Sf1 expression 33. Our findings demonstrated that the expression level of the ovarian β-catenin is markedly reduced in Wnt10a KO mice, with no significant changes in Sf1 expression. This contradiction between β-catenin and Sf1 requires further clarification. Previous studies indicated that β-catenin signaling activation could facilitate the effects of FSH on ovarian follicular cells 14. FSH binds to its receptor and activates downstream signals to promote the transactivation of Cyp19a1 by β-catenin signaling and facilitates estrogen production in the ovary 34. Conditional deletion of β-catenin in mouse granulosa cells blocks the Cyp19a1-stimulating effects of FSH, thereby affecting estradiol production 35. In the present study, we found that Cyp19a1 mRNA and protein expression in granulosa cells was significantly decreased in Wnt10a KO mice, leading to increases in the circulating progesterone and testosterone levels, and decreases in the circulating estrogen levels.

The gonadotropins, FSH and LH, and their receptors, FSHR and Lhcgr, play a central role in governing reproductive competency or fertility 1, 2. Disruptions of gonadotropins and/or their receptors can induce infertility. Lhcgr is a member of G protein-coupled receptor, which is crucial for female fertility. Lhcgr is expressed in the follicular theca cells, mature granulosa cells, stromal cells, and luteinized cells within the ovary. Activation of Lhcgr by LH promotes androgen production in theca cells, thereby providing the substrate for conversion to estrogen by FSH-induced activation of Cyp19a1 in granulosa cells. Activation of Lhcgr is also important for oocyte maturation, successful ovulation, and progesterone production in the corpus luteum 35. Deletion of Wnt signaling downregulated expression of Lhcgr within the ovary 36, corresponding with our findings. We detected no significant changes in the ovarian FSHR mRNA expression levels, and the circulating levels of FSH and LH in Wnt10a KO mice. The relationship between the hypothalamic-pituitary-gonadal axis and Wnt10a remains to be determined. Our findings indicated that Wnt10a may be dispensable for gonadotropin synthesis *in vivo*. Further studies are needed to explore the relationship among the Wnt signaling pathway, GnRH, gonadotropins, and their receptors.

In conclusion, our findings demonstrate that Wnt10a is required for female fertility through β-catenin and Cyp19a1 pathways to maintain the normal functions of the ovary and uterus, a regular estrous cycle, and the dynamic balance of the steroid hormones.

## Figures and Tables

**Table 1:** Expression of genes related to female fertility in ovarian tissue from WT and Wnt10a KO mice

Gene symbol	Gene ID	Fold change KO/WT	Gene description
Wnt10a	NM_009518	0.06	wingless related MMTV integration site 10a
Cyp19a1	NM_001312893.1	0.12	cytochrome P450, family 19, subfamily a, polypeptide 1
Esr1	NM_001302532.1	1.13	estrogen receptor 1 (alpha)
Esr2	NR_104386.1	1.25	estrogen receptor 2 (beta)
Ar	NM_013476.4	0.72	androgen receptor
Fshr	NM_013523.3	0.54	follicle stimulating hormone receptor
Fshb	NM_008045.3	1.19	follicle stimulating hormone beta
Lhcgr	NM_013582.2	0.34	luteinizing hormone/choriogonadotropin receptor
Lhb	NM_008497.2	1.23	luteinizing hormone beta
Creb1	NM_009952.2	1.22	cAMP responsive element binding protein 1
Nr5a1	NM_001316687.1	0.69	nuclear receptor subfamily 5, group A, member 1
